# Stimulating intestinal GIP release reduces food intake and body weight in mice

**DOI:** 10.1016/j.molmet.2024.101945

**Published:** 2024-04-21

**Authors:** Jo E. Lewis, Danae Nuzzaci, Paula-Peace James-Okoro, Mireia Montaner, Elisabeth O'Flaherty, Tamana Darwish, Marito Hayashi, Stephen D. Liberles, David Hornigold, Jacqueline Naylor, David Baker, Fiona M. Gribble, Frank Reimann

**Affiliations:** 1 Institute of Metabolic Science-Metabolic Research Laboratories & MRC-Metabolic Diseases Unit, University of Cambridge, Cambridge, UK; 2 Howard Hughes Medical Institute, Department of Cell Biology, Harvard Medical School, Boston, MA, USA; 3 Cardiovascular, Renal and Metabolic Diseases, BioPharmaceuticals R&D, AstraZeneca, Cambridge, UK; 4 Cardiovascular, Renal and Metabolic Diseases, BioPharmaceuticals R&D, AstraZeneca, Gothenburg, Sweden

**Keywords:** GIP, GLP-1, Obesity, Diabetes, Enteroendocrine cells, Feeding behaviour

## Abstract

**Objective:**

Glucose dependent insulinotropic polypeptide (GIP) is well established as an incretin hormone, boosting glucose-dependent insulin secretion. However, whilst anorectic actions of its sister-incretin glucagon-like peptide-1 (GLP-1) are well established, a physiological role for GIP in appetite regulation is controversial, despite the superior weight loss seen in preclinical models and humans with GLP-1/GIP dual receptor agonists compared with GLP-1R agonism alone.

**Methods:**

We generated a mouse model in which GIP expressing K-cells can be activated through hM3Dq Designer Receptor Activated by Designer Drugs (DREADD, GIP-Dq) to explore physiological actions of intestinally-released GIP.

**Results:**

In lean mice, Dq-stimulation of GIP expressing cells increased plasma GIP to levels similar to those found postprandially. The increase in GIP was associated with improved glucose tolerance, as expected, but also triggered an unexpected robust inhibition of food intake. Validating that this represented a response to intestinally-released GIP, the suppression of food intake was prevented by injecting mice peripherally or centrally with antagonistic GIPR-antibodies, and was reproduced in an intersectional model utilising Gip-Cre/Villin-Flp to limit Dq transgene expression to K-cells in the intestinal epithelium. The effects of GIP cell activation were maintained in diet induced obese mice, in which chronic K-cell activation reduced food intake and attenuated body weight gain.

**Conclusions:**

These studies establish a physiological gut-brain GIP-axis regulating food intake in mice, adding to the multi-faceted metabolic effects of GIP which need to be taken into account when developing GIPR-targeted therapies for obesity and diabetes.

## Highlights

• Dq-DREADD activation of intestinal K-cells increases GIP to post-prandial levels.
• K-cell stimulation improves glucose tolerance and reduced food intake in mice.
• Centrally injected GIPR antibodies block effects of K-cell activation on food intake.
• Chronic K-cell activation reduces food intake and body weight in diet-induced obesity.

## 1 Introduction

Glucose-dependent insulinotropic peptide (GIP) is secreted from the gut in response to nutrient ingestion and enhances meal-stimulated insulin secretion in a glucose dependent manner by activating its cognate receptor (GIPR) in pancreatic beta cells [1,2]. GIP is well recognised as an incretin and regulates blood glucose through both its insulinotropic and glucagonotropic actions in the pancreas [3]. For many years, GIP was neglected as a therapeutic target compared with GLP-1, as it was relatively ineffective at stimulating glucose dependent insulin release in patients with type 2 diabetes (T2D) when infused continuously [4]. However, new GIPR/GLP1R dual agonists in clinical trials have demonstrated superior outcomes (improved blood glucose control and body weight loss) compared with GLP1R agonism alone [5]. These results have rekindled interest in developing treatments for T2D and obesity based on the GIP-brain-pancreatic axis. However, many major questions regarding the physiology of GIP remain unanswered.

Despite longstanding recognition of GIP as an incretin, debate continues as to whether stimulation or inhibition of GIPR signalling would be the more effective approach for the treatment of metabolic disease. A number of key studies have suggested that GIP might promote weight gain: global germline GIPR knockout mice on a HFD display reduced body weight and maintain insulin sensitivity, whilst in patients with type 2 diabetes (T2D), the insulinotropic effect of GIP is impaired [4,6]. Indeed, GIPR antagonists improve body weight and glucose homeostasis in mice and non-human primates and enhance leptin's anorectic effect in high fat diet (HFD)- induced obese mice [7,8]. However, other studies have highlighted beneficial metabolic effects of raising GIP levels. Although GIP itself has a short half-life due to inactivation by dipeptidyl peptides 4 (DPP4), longer acting GIP analogues have been developed that are resistant to DPP4 inactivation (e.g. D-Ala2-GIP [9], or further stabilised by addition of an acyl side chain (e.g. acyl-GIP [10]) which, by analogy with long-acting GLP-1R peptide agonist, promotes albumin binding. Acyl-GIP decreases food intake and body weight in WT and *Glp1r* knockout (KO) mice, but not in global *Gipr* KO mice [10]. More recently it was shown that CNS GIPR signalling, especially in GABAergic neurons, is relevant to reductions in food intake [[11], [12], [13]]. D-Ala2-GIP by contrast has been reported not to affect food intake in mice [14,15], despite activating GABAergic neurons in the AP also implicated for the action of acyl-GIP [14]. Regardless, dual agonists targeting both GLP1R and GIPR decrease body weight and improve glucose tolerance in animal models of obesity and T2D in mice, non-human primates and obese patients with and without T2D [5,[16], [17], [18], [19]] with greater efficacy than GLP1R agonism alone. However, GIPR expressing neurons are likely to express other factors that alter food intake, whilst acyl-GIP differs in potency and pharmacokinetics from the native peptide. Furthermore, it is unclear whether these actions reflect physiological or pharmacological action.

To extend our understanding of the physiology of endogenous gut-derived GIP, we generated a GIP-Dq mouse model in which activation of a floxed hM3Dq-DREADD was achieved by Cre-recombinase expressed under the GIP promoter. Activation of GIP-Cre::hM3Dq cells in this model using CNO increased plasma GIP and improved glucose tolerance as expected, but also reduced food intake under a range of feeding paradigms. This suppression of feeding was surprising because we had earlier studied an intersectional model in which hM3Dq-DREADD expression was restricted to K-cells in the intestinal epithelium, and had not identified any feeding phenotype [20]. Further studies following up the finding of food intake suppression in the GIP-Dq model revealed that it was GIPR-dependent and was repeatable under a range of feeding paradigms, including in diet-induced obese (DIO) mice, and was also observed in the gut-restricted intersectional model. The results highlight a role for gut-derived GIP in the control of food intake.

## 2 Methods

### 2.1 Animal studies

All experiments were performed under the UK Home Office project licence PE50F6065 in accordance with the UK Animals (Scientific Procedures) Act, 1986 and approved by the University of Cambridge Animal Welfare and Ethical Review Body. All mice were from a C57BL/6 background and were group-housed and maintained in individual ventilated cages with standard bedding and enrichment in a temperature and humidity controlled room on a 12 h light:dark cycle (lights on 7:00) with ad libitum (AL) access to food (standard diet [std diet], Scientific Animal Food Engineering or 45% HFD) and water unless otherwise stated. For the chronic DCZ study, animals drinking water was treated with the DREADD ligand (at 100 μg/kg, or vehicle).

### 2.2 Generation of mouse models

To enable chemogenetic stimulated secretion of GIP, GIP-Cre mice [21] were crossed with with Rosa26-fxSTOPfx-hM3Dq reporter mice [22] or with Vil1-p2a-FlpO & FL-hM3Dq positive mice, creating intestinally restricted (GIP-Dq^INT^) hM3Dq expression [20].

### 2.3 Oral gavage, blood sampling and hormone measurement

Mice were fasted overnight (<16 h). Mice were gavaged with 200ul liquid Ensure. All blood samples were collected by capillary tubes via the tail vein in free-moving, conscious animals. Blood samples were placed immediately on ice, plasma collected post centrifugation and stored at −80 °C until required. Circulating hormones were measured via ELISA (MesoScale Discovery, insulin, total GLP-1 and PYY assays, UK) at Core Biochemical Assays Laboratories, Cambridge, UK. GIP was measured via ELISA as per the manufacturer's instructions (Millipore, UK).

### 2.4 Glucose tolerance tests

Mice were fasted overnight (<16 h). At the zero timepoint, glucose was administered at 2 g/kg body weight ip and either vehicle (VEH) or CNO was delivered ip contralaterally. Blood glucose was measured via the tail vein at just before (“0”) and 15-, 30-, 60-, 90- and 120-minutes post-administration (circa 5ul, Accu-Chek, UK). A minimum duration of 72 h between testing was employed. For the GIPR antagonism studies, animals received either an isotype control antibody or GIPR blocking antibody (Gipg013)^17^ at 19.2 mg/kg sc 48 h prior to intervention in a non-crossover design.

### 2.5 Food intake

Food intake studies were performed in a cross-over design, on age matched groups, a minimum of 72 h apart. Animals were singly housed prior to the experiment and fasted for 2 h. Mice were administered vehicle (VEH), CNO (1 mg/kg body weight, ip), or [D-Ala^2^]-GIP (300 nmol/kg body weight, ip). Food intake was measured at the timepoints indicated. For the fast-refeed experiment, animals were fasted overnight (<16 h) prior to presentation of the diet for 1hr. For the highly palatable meal (HPM) studies, animals were adapted to the appearance of liquid Ensure, at the onset of the dark phase, for 2 weeks (5 times per week), prior to intake measurement. For the antagonism studies, animals were treated with CCKR (Devazepide) and Y2R antagonist (JNJ-31020028) 30mins prior to intervention (as previous, [23]). Antagonism of the GLP-1 and GIP receptors was achieved with antibodies Gipg013(24) and Glp1R0017(25), respectively, at 19.2 mg/kg sc 48 h prior to intervention.

### 2.6 Metabolic cages

Animals were singly housed (for 5 days) and acclimated to metabolic cages prior to study and data collection. Oxygen consumption and carbon dioxide production were determined using an indirect calorimetry system (Promethion, Sable Systems, Las Vegas, NV). The system consisted of 16 metabolic cages (similar to home cages), equipped with water bottles and food hoppers connected to load cells for continuous monitoring, in a temperature and humidity-controlled room. The respiratory exchange ratio (RER) and energy expenditure (via the Weir equation) were calculated, whilst ambulatory activity was determined simultaneously. Raw data were processed using ExpeData (Sable Systems). Animals were exposed to standard chow or a HFD during metabolic assessment (as indicated). Animals were treated with VEH or CNO (as previous) at the onset of the dark phase (19:00).

### 2.7 Stereotaxic surgery

Mice, under isoflurane anaesthesia and receiving Metacam prior to surgery, were stereotactically implanted with a temporary guide cannula (Plastics One) positioned above the third ventricle (A/P −1.0 mm D/V −4.7  mm M/L 0.0 mm from bregma). Bevelled stainless steel injects (33 gauge, Plastics One) extending 1 mm from the tip of the guide were used for injections. For the GIPR monoclonal antibody antagonist studies, animals received an isotype control antibody or GIPR (1ug/100ul, at 50 nl/min). Mice were allowed 3 days recovery prior to testing. Whilst cannula tracts were assessed in every mouse postmortem and were consistent with ventricular targeting, due to the transient placement of the injection cannulas only during the antibody injection we were unable to confirm ventricular targeting with dye injection at the end of the experiment after behavioural assessment; data from all mice were included in the analysis.

### 2.8 Immunohistochemistry

Tissues were collected as previously described [26]. Tissue was stained overnight with primary antisera (Table 1) before incubation with appropriate secondary antisera (Table 1). Slides were imaged using an Axioscan Z1 slide scanner (Zeiss) and confocal microscope (Leica TCS SP8 X). For c-Fos studies, 2–6 sections per mouse at the level of the hypothalamus (bregma −1.58 to −2.3 mm) and hindbrain (bregma −7.2 to −7.76 mm) were counted bilaterally and averaged across sections for each mouse. Images were analysed in ImageJ.

**Table 1:** Antibodies.

Target (Cat. No)	Primary	Secondary (AlexaFluorophore,ThermoScientific, UK)
GFP (ab5450)	1:2000(chicken, Abcam, UK)	Goat anti-chicken 488 (A32931)
cFOS (226017)	1:2000(goat, SYSY, USA)	Goat anti-chicken 555 (A21437)

### 2.9 Statistical analysis

Data were plotted using GraphPad Prism 9 software. Statistical analysis was performed by t-test (paired), one way ANOVA, two-way ANOVA (time x treatment) with post hoc comparisons (where appropriate) and ANCOVA (using body weight as a covariate) as indicated. N represents biological replicates. Sample size was computed based on pilot data and previously published data. Data are presented as mean ± SEM and probabilities of p < 0.05 were considered statistically significant in all tests.

## 3 Results

### 3.1 Chemogenetic activation of GIP-Cre::hM3Dq cells elevates plasma GIP in the physiological range and improves glucose tolerance

**Figure 1:**
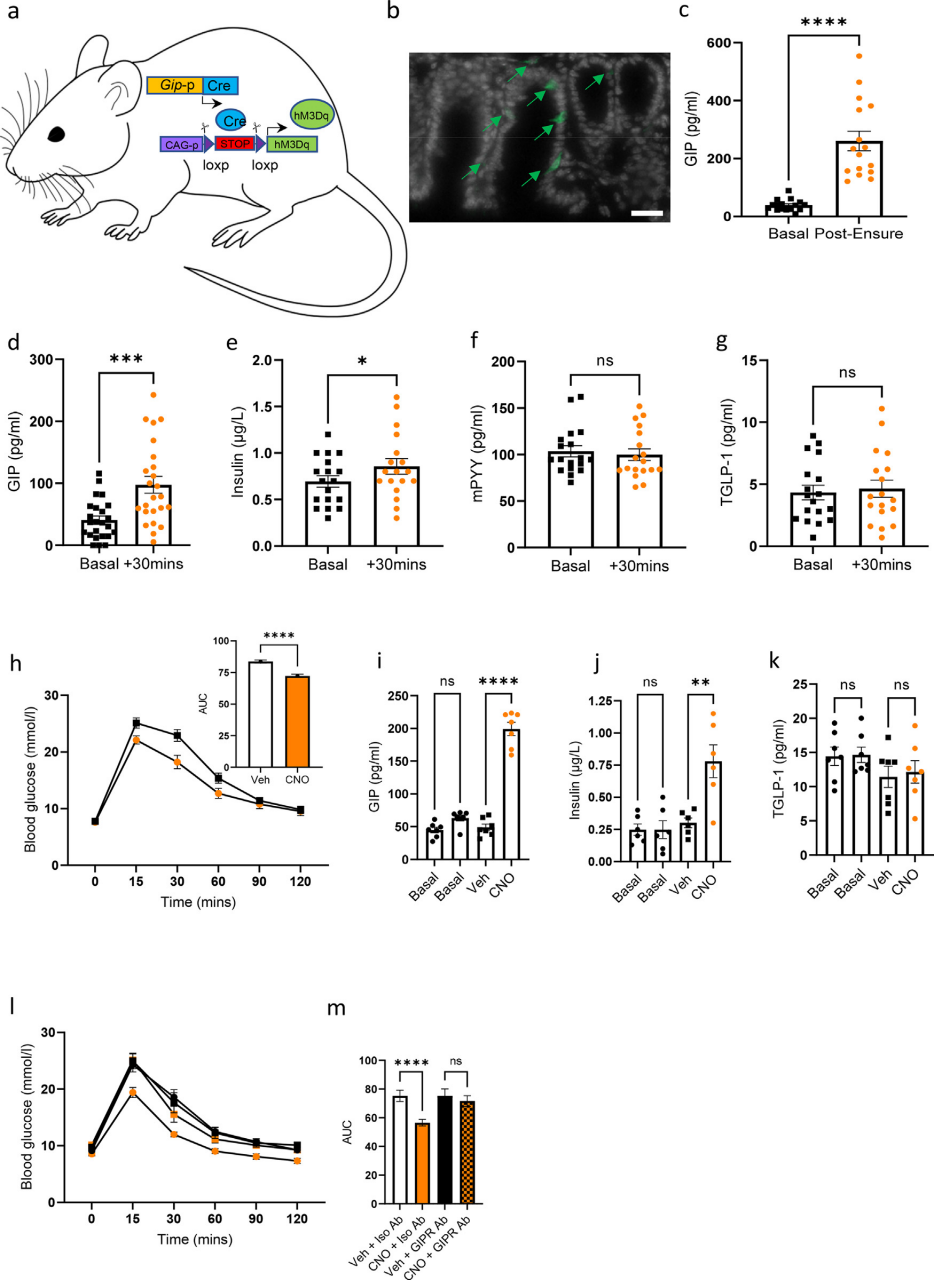
**GIP-Cre::hM3Dq activation significantly improves glucose tolerance.** (A) Schematic for the GIP-Dq mouse model. (B) Representative section from the small intestine of GIP-Dq mice demonstrating Dq (green) and DAPI (white) expression. Scale bar – 100um. (C) Plasma GIP of WT mice receiving oral gavage of liquid Ensure, a mixed meal. (D–G) Plasma (D) GIP, (E) insulin, (F) mPYY and (G) total GLP-1 (TGLP-1) of GIP-Dq mice treated with CNO (1 mg/kg BW ip). (H) ipgtt (2 g/kg BW glucose, admin of VEH or CNO (at 1 mg/kg ip, delivered contralaterally to glucose at time 0) with AUC (inset) (n = 16–24 per group). (I–K) Plasma (I) GIP (one-way ANOVA: effect of treatment F_(3,24)_ = 133.3, p < 0.0001. Post hoc p < 0.0001), (J) insulin (effect of treatment F_(3,20)_ = 10.95, p < 0.0001. Post hoc p = 0.0017) and (K) TGLP-1 (effect of treatment F_(3,24)_ = 1.275, p = 0.3053) at basal and +15 mins post glucose (as previous). Animals were subsequently pre-treated with a GIPR monoclonal antibody antagonist or isotype control antibody 48 h prior to (L) ipgtt (as previous) and (M) AUC (n = 7 per group, effect of treatment F_(3,24)_ = 38.73, p < 0.0001. Post hoc p = 0.0001). Values are presented as group mean ± SEM. ∗p < 0.05, ∗∗∗p < 0.001 and ∗∗∗∗p < 0.0001 by paired students T test (C-H[inset]) and one-way ANOVA (I-K, M[inset]).

Generation of the mouse model (abbreviated hereafter as GIP-Dq) is depicted in Figure 1A. First, we demonstrated that Dq expression in the intestine was limited to a subset of enteroendocrine cells (Figure 1B). Gavaging wild type (WT) mice with liquid Ensure, a highly palatable mixed meal, resulted in a significant increase in plasma GIP (Figure 1C). Activation of GIP-Cre::hM3Dq cells by treating GIP-Dq mice with CNO (1 mg/kg BW) also resulted in a significant increase in plasma GIP at 30 min, with a concurrent increase in plasma insulin (Figure 1D,E). There was no observable increase in PYY or total GLP-1 (TGLP-1) demonstrating the functional specificity of the Dq to GIP expressing cells (Figure 1F,G). To further validate the model, we conducted an intraperitoneal glucose tolerance test (ipgtt) and observed a significant improvement in glucose tolerance when GIP-Dq mice were treated with CNO, associated with a significant increase in plasma GIP and insulin, with no change in TGLP-1 (Figure 1H(plus inset)–K). To demonstrate that the improved glucose tolerance was attributable to GIP, mice were pre-treated with either a monoclonal GIPR antagonistic antibody or an isotype control revealing that antagonising GIPR abolished the effects of GIP-Cre::hM3Dq activation on ip glucose tolerance (Figure 1L,M), concomitantly excluding significant direct driving of hM3Dq in pancreatic alpha or beta cells, as pancreatic *Gip* expression has been reported by others, but was not observed in our hands [24,27].

### 3.2 Chemogenetic activation of GIP-expressing cells reduces food intake

**Figure 2:**
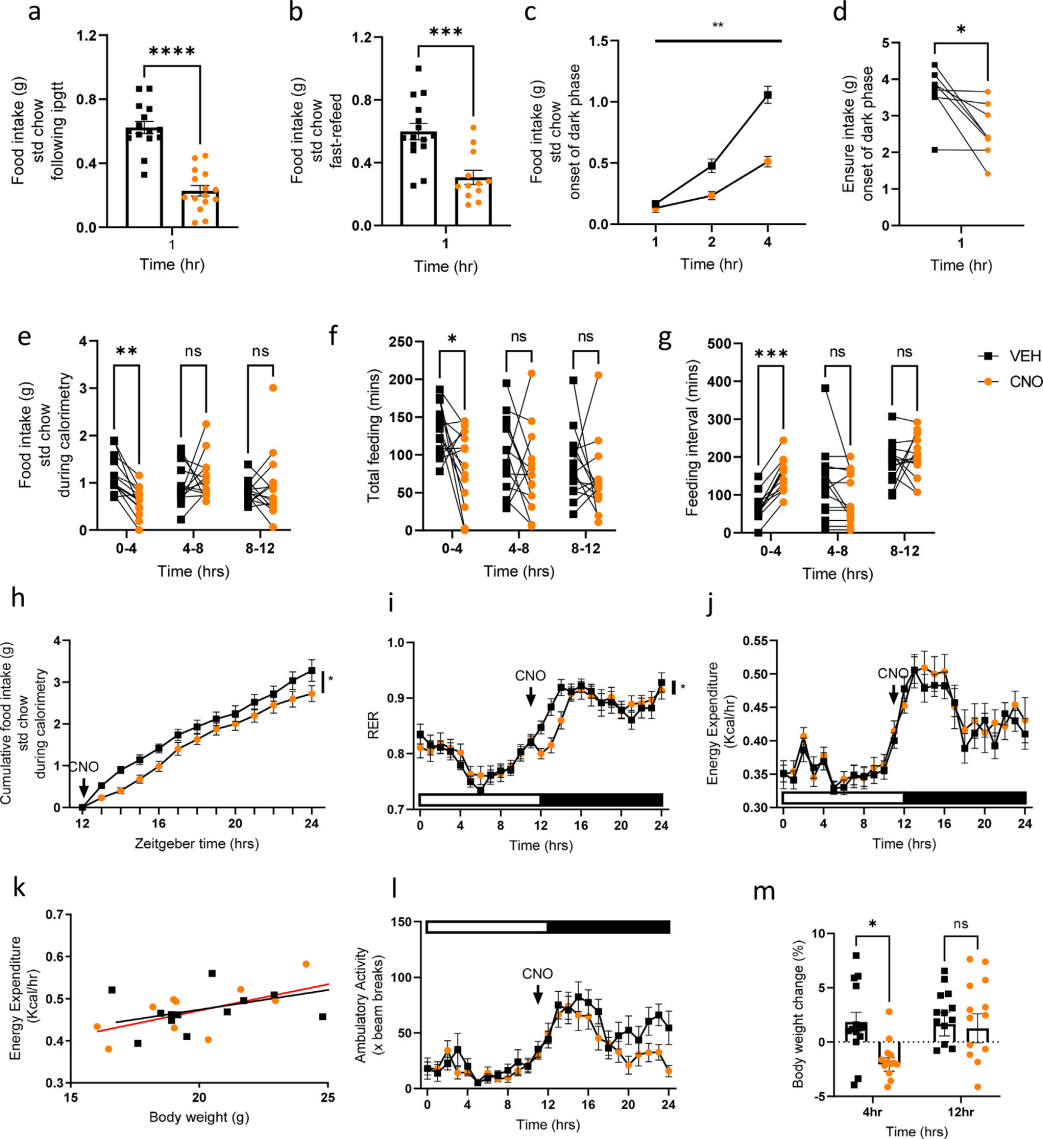
**GIP-Cre::hM3Dq activation significantly reduces food intake**. (A) Food intake of GIP-Dq mice 1hr post ipgtt (n = 15 per treatment). (B) Overnight fast (16 h)-refeeding(1hr) food intake of GIP-Dq mice treated with VEH/CNO (n = 12–15 per treatment). (C) Food intake of ad lid fed GIP-Dq mice treated with VEH/CNO (at 1 mg/kg BW ip) at the onset of the dark phase (n = 5–6 per treatment, treatment F_(1,8)_ = 20.66, p = 0.0042, time F_(1.628, 13.03)_ = 180.6, p < 0.0001, interaction F_(2,16)_ = 28.31, p < 0.0001). (D) HPM intake of ad lib fed mice treated with CNO at the onset of the dark phase (n = 8 per treatment). (E) Food intake (interaction F_(2,50)_ = 5.578, p = 0.0065. Post hoc p = 0.001), (F) Total meal duration (treatment F_(1,26)_ = 6.007), p = 0.0213, time F_(1.933,50.27)_ = 4.181, p = 0.022. Post hoc p = 0.0225 (G) Inter-meal interval (interaction F_(2,76)_ = 5.269, p = 0.0072, time F_(1.659,63.02)_ = 16.88, p < 0.0001). Post hoc p = 0.0001), (H) Cumulative food intake (treatment F_(1,25)_] = 4.775, p = 0.0385, time F_(2.093,52.33)_ = 178.6, p < 0.0001, (I) RER (interaction F_(24,610)_ = 2.033, p = 0.0027, time F(_6.816,173.2)_ = 42.37, p < 0.0001), (J,K) Energy expenditure (time F_(9.234,234.7)_ = 25.67, p < 0.0001), (L) Ambulatory activity (time F_(7.607, 193.3)_ = 13.30, p < 0.0001) and (M) body weight change of ad lib fed GIP-Dq mice treated with VEH/CNO at the onset of the dark phase (n = 14 per treatment, interaction F_(1,26)_ = 4.242, p = 0.0496. Post hoc p = 0.0189). Values are presented as group mean ± SEM. ∗p < 0.05, ∗∗p < 0.01, and ∗∗∗p < 0.001 by paired (A, D) and students T test (B) and two-way ANOVA (C, E-J, L,M) and ANCOVA (body weight as covariate, K).

We routinely measure food intake in the 1hr after ipgtts and to our surprise food intake was significantly reduced in the CNO arm of this cross-over study (Figure 2A). In naïve GIP-Dq mice, chow intake was also reduced during the 1hr refeed window following an overnight fast (Figure 2B). CNO administration at the onset of the dark phase similarly and significantly reduced food intake (Figure 2C), as was intake of a highly palatable meal (HPM, liquid Ensure) in mice adapted to its appearance at the onset of the dark phase (Figure 2D). To further explore this robust feeding phenotype, we placed animals in metabolic cages. Upon GIP-Cre::hM3Dq activation with CNO at the onset of the dark phase, GIP-Dq mice demonstrated significantly reduced food intake, associated with reduced feeding times and an increase in intervals between feeding (Figure 2E–G). Corresponding with the lower cumulative food intake (Figure 2H) was an apparent significant reduction in RER (Figure 2I), as often observed when animals reduce carbohydrate intake and thus delay the switch from fat oxidation. Together, these results suggest that acute release of GIP from the small intestine reduces food intake. There was no effect of GIP-Cre::hM3Dq activation on energy expenditure (measured via two-way ANOVA or ANCOVA using body weight as a covariate) or ambulatory activity (Figure 2J-L). Animals demonstrated an initial reduction in body weight, likely due to reduced intestinal contents at such an early time point, which was normalised at 12 h post treatment (Figure 2M).

The robust suppression of food intake in GIP-Dq mice was surprising in the light of recently reported contradictory findings using the same GIP-Cre strain in a genetic intersectional model that restricted hM3Dq expression to the intestinal epithelium [20], and led us to consider possible explanations for the discrepancy. One idea we considered was whether there might be a cell population in the CNS that was directly activated by CNO in GIP-Dq mice but not in the intersectional model. Extensive analyses by immunofluorescent microscopy of the brains of GIP-Cre mice crossed with Cre-dependent reporter strains, however failed to identify any GIP-Cre-dependent fluorescent reporter activation in the CNS (Supplementary Figs. 1a–h). We also failed to detect any GIP-Cre-dependent fluorescence in the pancreas (Supplementary Figs. 1i and j).

Our next approach was to re-create the intersectional model (Gip-Cre x Vil1-p2a-FlpOGIP-Dq (GIP-Dq^INT^)). In the Cambridge-UK facility, GIP-Dq^INT^ mice treated with CNO exhibited improved glucose tolerance and reduced food intake at the onset of the dark phase in ad lib fed mice, reproducing the phenotype of global GIP-Dq mice. As in GIP-Dq mice, this was associated with a significant increase in plasma GIP (Supplementary Figs. 2a–c). The results indicate that activation of intestinal K-cells underlies the improved glucose tolerance and feeding reduction in both global GIP-Dq and GIP-Dq^INT^ mice.

### 3.3 Food intake reduction is sensitive to both peripherally and centrally administered GIPR-blocking antibody

To demonstrate that the effect on food intake was specific to GIP rather than known co-secreted hormones we repeated the experiment after pre-treating GIP-Dq mice with CCK1R, NPY2R or GLP1R antagonists [25]. These did not alter the feeding phenotype observed upon GIP-Cre::hM3Dq activation at the onset of the dark phase (Supplementary Figs. 2d–f). By contrast, peripheral pre-treatment with the GIPR antagonistic antibody abolished the inhibition of feeding at the onset of the dark phase, both in the fast refeed paradigm and in animals adapted to the appearance of a HPM at the onset of the dark phase (Supplementary Figs. 2g–i). Interestingly, treatment of WT mice with the GIPR antagonist significantly increased the intake of the HPM (Supplementary Fig. 2j), and whilst plasma TGLP-1 and insulin were not affected by the treatment, blood glucose in the post-prandial state was significantly elevated (Supplementary Fig. 2k-m).

**Figure 3:**
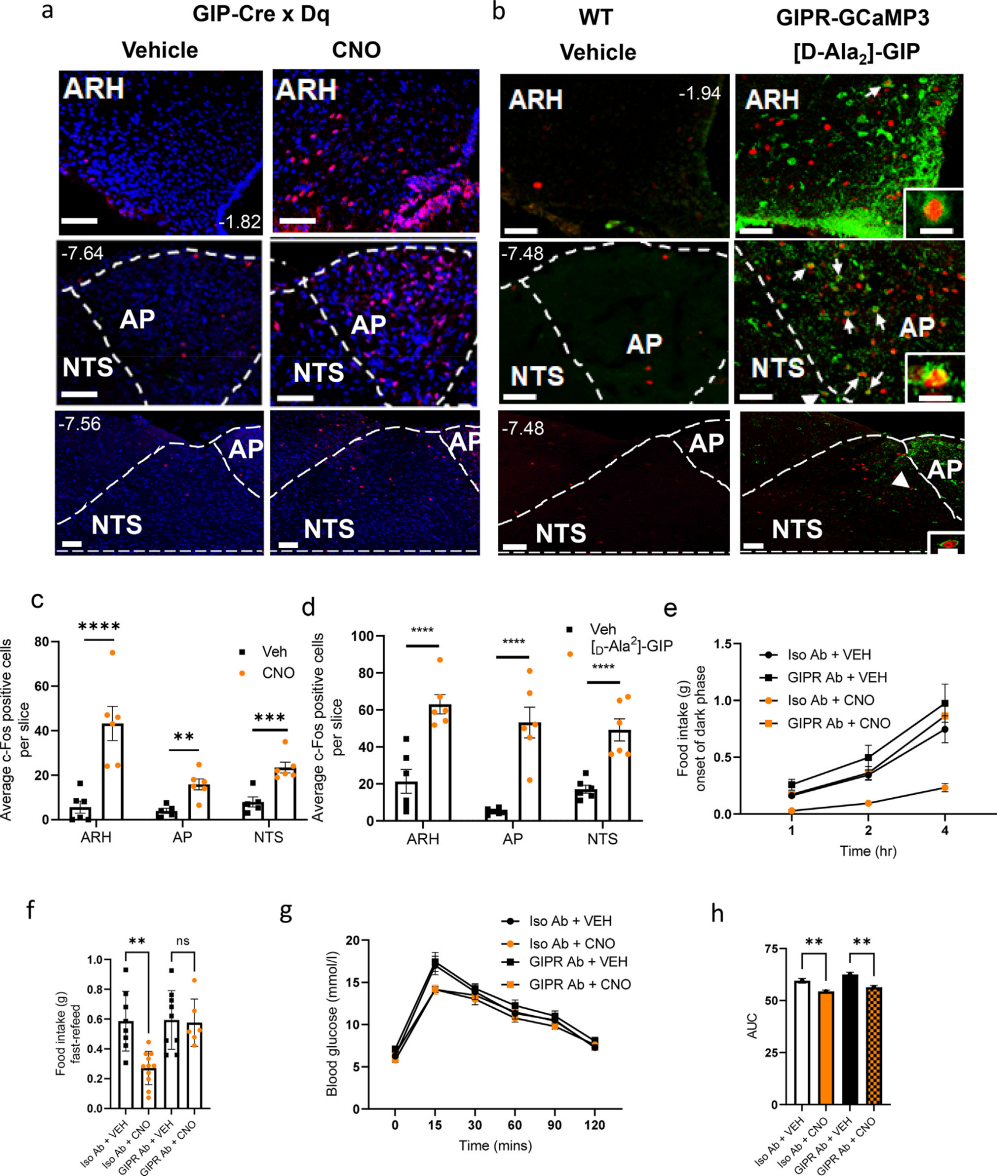
**Central GIPR antagonism abolishes the effect on food intake but not glucose tolerance.** Representative cfos staining of the ARC, AP and NTS of (A) GIP-Dq mice treated with VEH/CNO (at 1 mg/kg BW ip, n = 6 per group) and (B) WT/GIPR-Cre::GCaMP3 mice treated with VEH/[D-Ala2]-GIP; scale bars represent 100 μm and numbers at the top refer to Bregma. (C,D), quantification of conditions shown in a and b, respectively showing average c-Fos positive cells per section. Each point represents data from an individual mouse. (E) Food intake (at the onset of the dark phase) of ad lib fed GIP-Dq mice receiving ICV pre-treatment with IsoAb/GIPR Ab antagonist (n = 4–5 per group, interaction F(_6,26)_ = 7.174, p = 0.0001, time F_(1.116,14.51)_ = 137.5, p < 0.0001, treatment F_(1,13)_ = 9.690, p = 0.0013. Post hoc p = 0.030). (F) Food intake in fast/refeed paradigm (n = 8–11 per group, treatment F_(3,30)_ = 8.765, p = 0.0003. Post hoc p = 0.0019). (G) ipgtt (as previous) and (H) AUC (n = 3–6 per group, treatment F_(3,14)_ = 15.77, p < 0.0001. Post hoc p = 0.0079 and p = 0.0054 respectively). Values are presented as group mean ± SEM. ∗∗p < 0.01 by Students T test (C and D), two-way ANOVA (E) and one-way ANOVA (F, H).

As GIP has not been reported to influence afferent vagal nerve activity, the observed reduction in food intake after GIP-Cre::hM3Dq activation suggested a central target of GIP. Acyl-GIP has previously been shown to reduce food intake and increase c-Fos in hypothalamic feeding centres [12] and recently has been shown to engage GABAergic neurons in the brain stem [13]. Mirroring these findings, treating GIP-Dq mice with CNO was associated with increased c-Fos staining in the hypothalamus (arcuate nucleus ARH, dorsomedial hypothalamus DMH, ventromedial hypothalamus VMH and median eminence ME) and the brainstem (area postrema AP and nucleus tractus solitarius NTS) (Figure 3A,C). D-Ala2-GIP treatment of GIPR-Cre::GCaMP3 mice induced a similar fos-activity pattern (Figure 3B,D and Supplementary Figs. 3a–d), with the strongest overlap of fos and GIPR-Cre reporting in AP neurons, consistent with previous reports for exogenous GIPR-agonists [13,14]. Consistent with these similar patterns of neuronal activation, but in contrast to Costa et al. [14] we were able to observe a small, but significant inhibition of food intake upon application of D-Ala2-GIP at the onset of the dark phase in two independent cohorts (Supplementary Fig. 3e).

To further assess whether the reduction in food intake was centrally mediated, we determined the effect of central GIPR blockade by delivering the GIPR antagonist antibody via intracerebroventricular infusion, directed towards the third ventricle, in GIP-Dq mice. Central pre-treatment with the GIPR antagonistic antibody abolished the suppression of food intake at the onset of the dark phase and in the fast-refeed paradigm (Figure 3E,F). However, central GIPR antagonism did not alter glucose tolerance (Figure 3G,H).

### 3.4 In DIO mice the effects of GIP-Cre::hM3Dq activation are maintained and chronic chemogenetic activation reduces body weight gain

**Figure 4:**
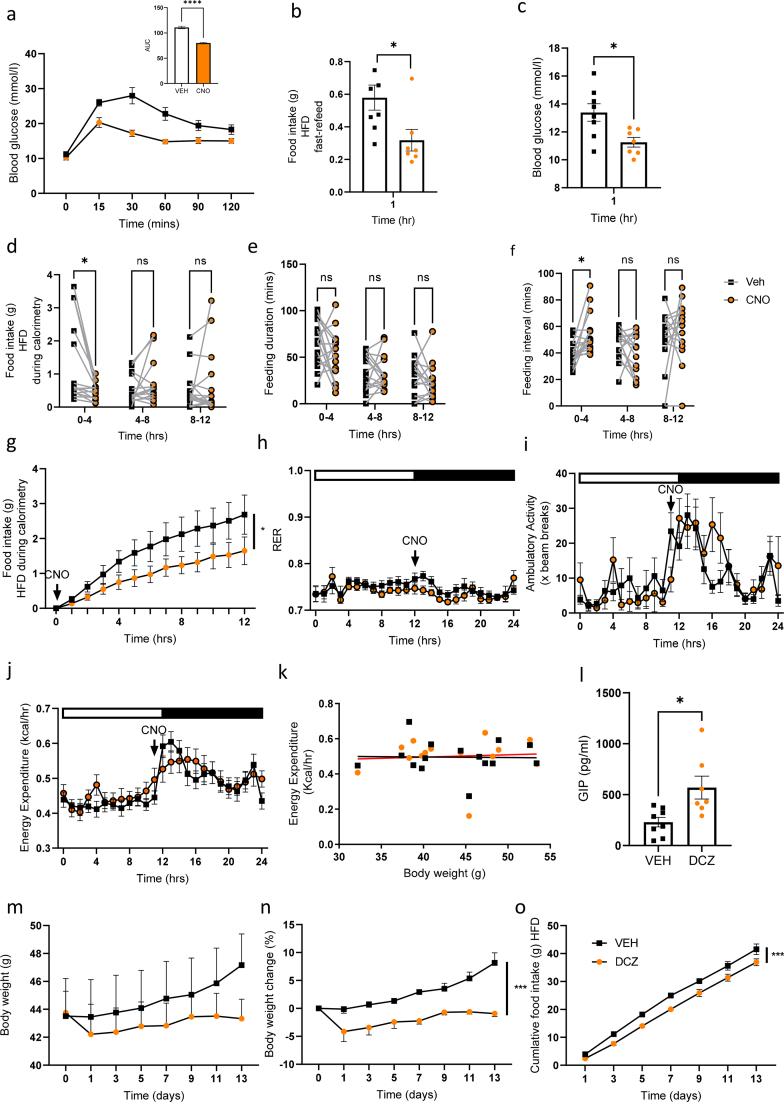
**The effects of GIP-Cre::hM3Dq activation are maintained in DIO mice.** (A) ipgtt (2 g/kg BW glucose, admin of VEH or CNO (at 1 mg/kg ip, delivered contralaterally to glucose at time 0) with AUC (inset) (n = 8 per group). (B) Fast-refeed (as previous) food intake of GIP-Dq mice following treatment with VEH/CNO (at 1 mg/kg BW ip, n = 7–8 per group). (C) Blood glucose in ad lib fed state following treatment with VEH/CNO (n = 7–8 per group). (D) Food intake (interaction F_(2,56)_ = 6.432, p = 0.0031. Post hoc p = 0.0221), (E) Total meal duration (F) Inter-meal interval (treatment F_(1,28)_ = 4.478, p = 0.0434. Post hoc p = 0.0436), (G) Cumulative food intake (interaction F_(12,336)_ = 2.026, p = 0.0215), (H) RER (time F_(6.486,147)_ = 4.453, p = 0.0002), (I) Ambulatory activity (time F_(5.666,132.7)_ = 7.531, p < 0.0001) and (J,K) Energy expenditure (time F_(4,692,109.5)_ = 21.30, p < 0.0001) of GIP-Dq mice treated with VEH/CNO at the onset of the dark phase in ad lib fed animals (n = 15 per treatment). (L) Plasma GIP, (M) Body weight (interaction F_(7,91)_ = 6.117 p < 0.0001), (N) Body weight change (interaction F_(7,78)_ = 2.435, p = 0.0261, treatment F_(1,13)_ = 23.30, p = 0.0003, time F_(2.596, 28.93)_ = 10.74, p = 0.0001) and (O) Cumulative food intake (treatment F_(1,13)_ = 25.65, p = 0.0002), time F_(1.607,20.89)_ = 443.8, p < 0.0001) of GIP-Dq mice treated chronically with DCZ via drinking water (n = 7–8 per group). Values are presented as group mean ± SEM. ∗p < 0.05, ∗∗p < 0.01, and ∗∗∗p < 0.001 by Students T test (A,B(inset)C, D) and two-way ANOVA (D-J, L-N) and ANCOVA (body weight as covariate, K).

To further assess the role of intestinal GIP, we fed GIP-Dq mice a HFD for 14 weeks (body weight = 43.2 ± 1.5 g) before assessing the outcome of chemogenetic stimulated GIP secretion. Activation of GIP-Dq significantly improved glucose tolerance as assessed by ipgtt (Figure 4A). Following a fast-refeed, food intake was significantly reduced (Figure 4B) and blood glucose in the fed state was significantly reduced (Figure 4C). To assess the microarchitecture of food intake patterns and whole-body physiology, we placed DIO animals in metabolic cages and treated with CNO at the onset of the dark phase. Treatment resulted in a significant reduction in food intake, and whilst no change in meal duration was apparent, there was a significant increase in the feeding interval time (Figure 4D–F). We note that these data are quite varied, with some animals in the control arm consuming unusual high amounts of food in the first 4 h – whilst we checked for unconsumed food in the cage, we cannot exclude a methodological error; the observed reduction in food intake and increase in the feeding interval in response to CNO are, however, consistent with the other data presented in Figure 4 acquired independently of the calorimetric chambers. Food intake was significantly reduced across the dark phase, but RER was unchanged (Figure 4G,H). Ambulatory activity and energy expenditure (as assessed by two-way ANOVA and ANCOVA, using body weight as a covariate), were also unchanged (Figure 4I–K). Finally, the effects of chronic activation of GIP-Cre::hM3Dq were assessed by the addition of the DREADD ligand deschloroclozapine (DCZ, with improved affinity and greater agonist potency for hM3Dq compared with CNO, and increased stability) to the drinking water [28]. Compared with vehicle treated mice, plasma GIP was significantly increased in the DCZ group in ad lib fed mice (Figure 4L). Body weight in the DCZ cohort was initially reduced and subsequent gain was attenuated, in parallel with significantly reduced food intake (Figure 4M–O). Taken together these results demonstrate a physiological role for intestinal GIP in glucose homeostasis and food intake via CNS GIPR signalling.

## 4 Discussion

Hormones from the gut that signal nutrient uptake and availability to the brain are key elements in the control of appetite [29]. GIP is secreted from the small intestine in response to nutrient ingestion and enhances meal-stimulated insulin secretion in a glucose dependent manner by activating its cognate receptor in pancreatic beta-cells [1,30]. Here we demonstrate that gavaging liquid Ensure, a mixed meal, results in a significant increase in plasma GIP in lean mice. In our GIP-Dq model, treatment with the DREADD ligand CNO significantly increased plasma GIP to a similar (and therefore physiological) level. It did so without altering PYY or GLP-1, demonstrating the specificity of the hM3Dq to GIP expressing cells.

Our data demonstrate the ability of GIP to improve glucose tolerance in lean and DIO mice, and to reduce food intake. The improvement of glucose tolerance likely reflects direct action of GIP on pancreatic beta cells, as this incretin effect was blocked by peripheral but not centrally administered GIPR-blocking antibodies. GIP-Cre::hM3Dq activation resulted in neural activation in the hypothalamus (ME, ARH, VMH and DMH) in addition to the AP and NTS in the hindbrain, as determined by c-Fos labelling, and likely underlying to the observed feeding phenotype. Fos-labelling of the AP and hypothalamic nuclei have previously been observed upon acute peripheral treatment with acyl-GIP [12,13]. Blockade of central GIPR via ICV infusion of a monoclonal antagonistic antibody, abolished the feeding phenotype associated with GIP-Cre::hM3Dq activation. These data are consistent with previous reports that *Gipr* is expressed in the hypothalamus and hindbrain, that chemogenetic activation of the hypothalamic and hindbrain GIPR population reduces food intake, and that centrally administered acyl-GIP decreases body weight and food intake via CNS GIPR in DIO mice [[11], [12], [13],^31^]. However, peripheral GIP has limited permeability into deeper regions of the hypothalamus due to the blood brain barrier, and our results do not enable us to conclude which neuronal populations were activated directly by GIP compared with activation arising secondary to GIP binding in distant brain areas. The limited overlap of fos staining with GIPR-Cre-labelled cells in the hypothalamus suggests that this might be dominated by signalling downstream of GIPR-activation at other sites, as from, for example, the area postrema, where a stronger overlap was observed.

The robust suppression of feeding in GIP-Dq mice was unexpected in the light of other studies which have shown only modest food intake suppression by GIP analogues in mouse models, and no effect on feeding in humans [10,32]. Our finding that food intake was also suppressed in the intestinally-restricted intersectional GIP-Dq model, when studied in the same animal facility as the global Gip-Dq mice, supports the idea that the feeding phenotype arose from stimulation of intestinal K-cells. We are unable to explain why our previous analysis of this intersectional model in a different facility did not identify a similar food intake phenotype, as the experimental conditions were not substantially different [20]. It is possible that the notably strong response to GIP-Cre::hM3Dq activation reported in the current study arose because of the simultaneous release from K-cells of additional factors such as gut hormones or small molecular transmitters, that synergised with GIP to enhance satiety or satiation. In a previous study we found that Dq-stimulation of L-cells in the distal colon and rectum which produce INSL5, GLP-1 and PYY (INSL5-Dq model), suppressed food intake through a pathway that was blocked by NPY2R inhibition, demonstrating that PYY release from the colon can reduce feeding [23]. By contrast in the GIP-Dq model, inhibitors of GLP1R, NPY2R and CCKR1 did not prevent the suppression of food intake. It is possible that stimulation of GIP-Cre::hM3Dq cells triggered local release of ATP or glutamate, triggering activity in the afferent vagus nerve, as reported previously for other enteroendocrine cell types [33,34]. High local GIP concentrations in the intestinal epithelium could also cause paracrine activation of enterochromaffin cells [35], triggering the release of serotonin which could contribute to food intake inhibition [20,36], although the effectiveness of centrally administered GIPR-blocking antibody suggests a direct action of GIP at the CNS. In both the GIP-Dq and INSL5-Dq models, the suppression of food intake triggered by CNO was transient, lasting only a few hours, due to the short half-lives of both CNO [37] and the secreted gut hormones [38].

The inhibitory effect on food intake of GIP-Cre::hM3Dq activation is also difficult to reconcile with literature suggesting that GIPR antagonism rather than agonism is beneficial for weight loss. Despite suggestions that some GIPR agonists might act as functional antagonists by promoting receptor internalisation, the ability of GIP-Cre::hM3Dq activation to reduce food intake and consequently body weight is unlikely to be driven by a functional reduction in GIPR signalling, as when lean animals were fed a HPM, GIPR antagonism significantly increased intake. Whilst we did not assess central *Gipr* expression, it has been previously shown that chronic central administration of acyl-GIP reduces food intake and body weight in DIO mice, without changes in central or peripheral *Gipr* expression [12]. Reduction in food intake by acyl-GIP was recently linked to activation of GABAergic neurons, likely including neurons in the AP [13]; whilst D-Ala2-GIP also activates GABAergic neurons in the AP, it was reported to have no effect on food intake by itself [14]. A possible explanation for these contrasting findings and our results might involve different pharmacokinetics and pharmacodynamics of GIPR-agonists. We speculate that intermittent activation, as implemented by the GIP-Cre::hM3Dq DCZ model unveils a physiological role of GIP signalling for food intake control that is missed with prolonged acting agonists, possibly due to receptor desensitisation; acyl-GIP retains potency, whereas D-ala2-GIP has a small effect, that was observed here, but not by others.

In summary, we demonstrate a physiological role for GIP in the regulation of food intake and body weight in mice. Importantly, we found this action was blocked by central pre-treatment with a GIPR antagonist, demonstrating the effect is specific to GIP and is centrally mediated. Whilst we cannot completely exclude a central source of GIP, which has previously been reported in rats [39,40], the fact that genetic intersectional K-cell restricted Dq activation phenocopied the results in global GIP-Dq mice, and our failure to observe any central GIP-Cre-dependent reporter activity in our mouse model, clearly demonstrates a role of intestinal K-cell secretion in the regulation of food intake in mice, important for our understanding of pharmacotherapies based on dual GLP-1/GIP agonism.

## CRediT authorship contribution statement

**Jo E. Lewis:** Writing – review & editing, Writing – original draft, Visualization, Supervision, Methodology, Investigation, Funding acquisition, Formal analysis, Data curation, Conceptualization. **Danae Nuzzaci:** Writing – review & editing, Methodology, Investigation, Formal analysis, Data curation. **Paula-Peace James-Okoro:** Writing – review & editing, Investigation, Formal analysis, Data curation. **Mireia Montaner:** Writing – review & editing, Methodology, Investigation, Formal analysis, Data curation. **Elisabeth O'Flaherty Rottenberger:** Writing – review & editing, Formal analysis, Data curation. **Tamana Darwish:** Writing – review & editing, Data curation. **Marito Hayashi:** Resources, Methodology. **Stephen D. Liberles:** Resources, Methodology. **David Hornigold:** Resources. **Jacqueline Naylor:** Resources. **David Baker:** Resources. **Fiona M. Gribble:** Writing – review & editing, Writing – original draft, Supervision, Resources, Project administration, Investigation, Funding acquisition, Formal analysis, Conceptualization. **Frank Reimann:** Writing – review & editing, Writing – original draft, Supervision, Resources, Project administration, Funding acquisition, Formal analysis, Conceptualization.

## Declaration of competing interest

The Gribble-Reimann lab has received funding from AstraZeneca and Eli Lilly & Company in the past and PPJO studentship is in partnership with AstraZeneca. DH, JN and DB are AstraZeneca employees.

## Data Availability

Data will be made available on request.
