# Supplementary material for: Stimulating intestinal GIP release reduces food intake and body weight in mice
Source: Mol Metab. 2024 Apr 21;84:101945. doi: 10.1016/j.molmet.2024.101945 (PMC11070708; doi:10.1016/j.molmet.2024.101945)
Supplement: Multimedia component 1 [file mmc1.pptx]

## Slide 1
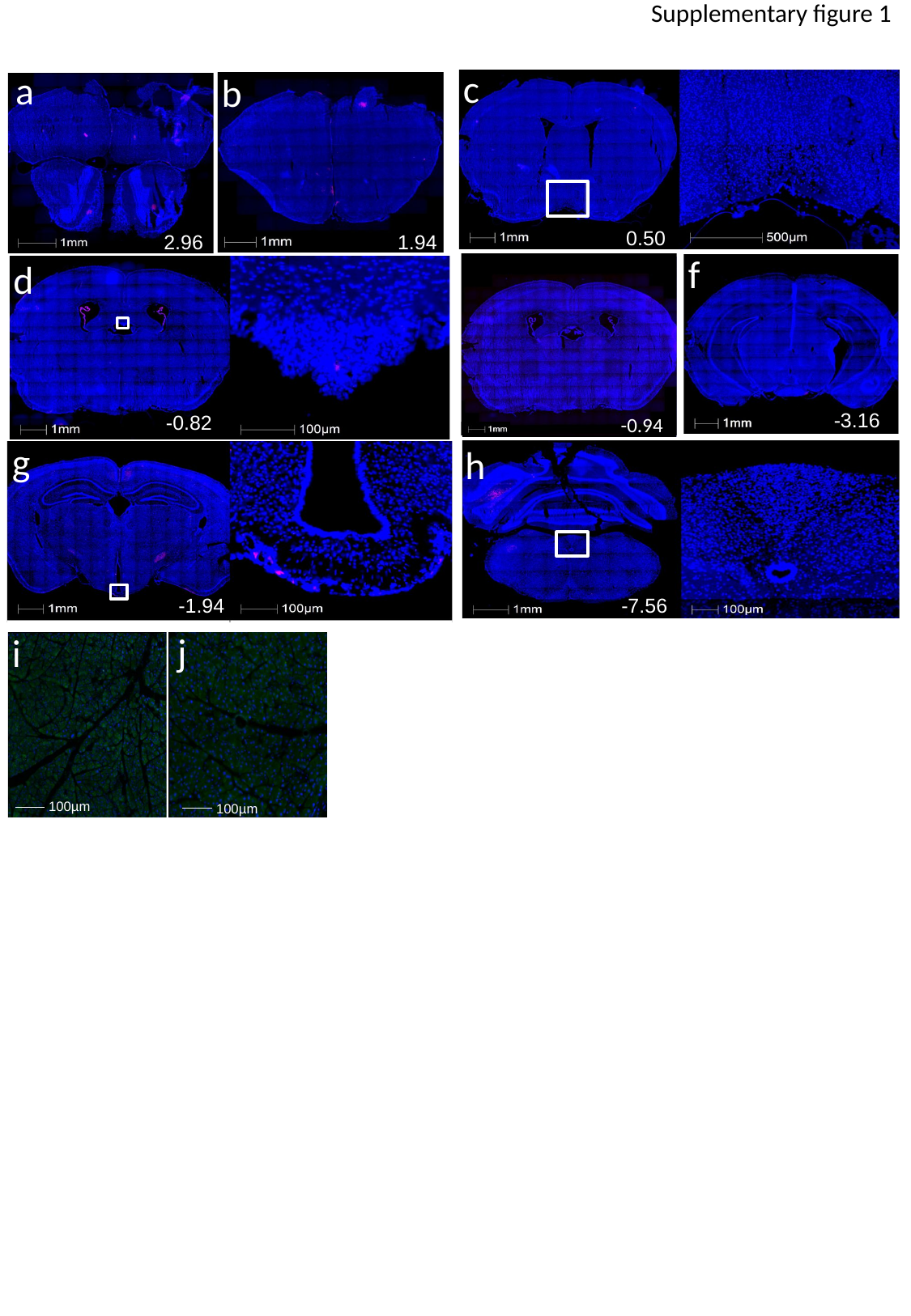

Supplementary figure 1
c
0.50
a
2.96mm
b
1.94
B
0.50mm
f
-3.16
d
-0.82
e
-0.94
-1.82mm
g
h
-7.56
-0.94
-1.94
j
i
100µm
100µm

## Slide 2
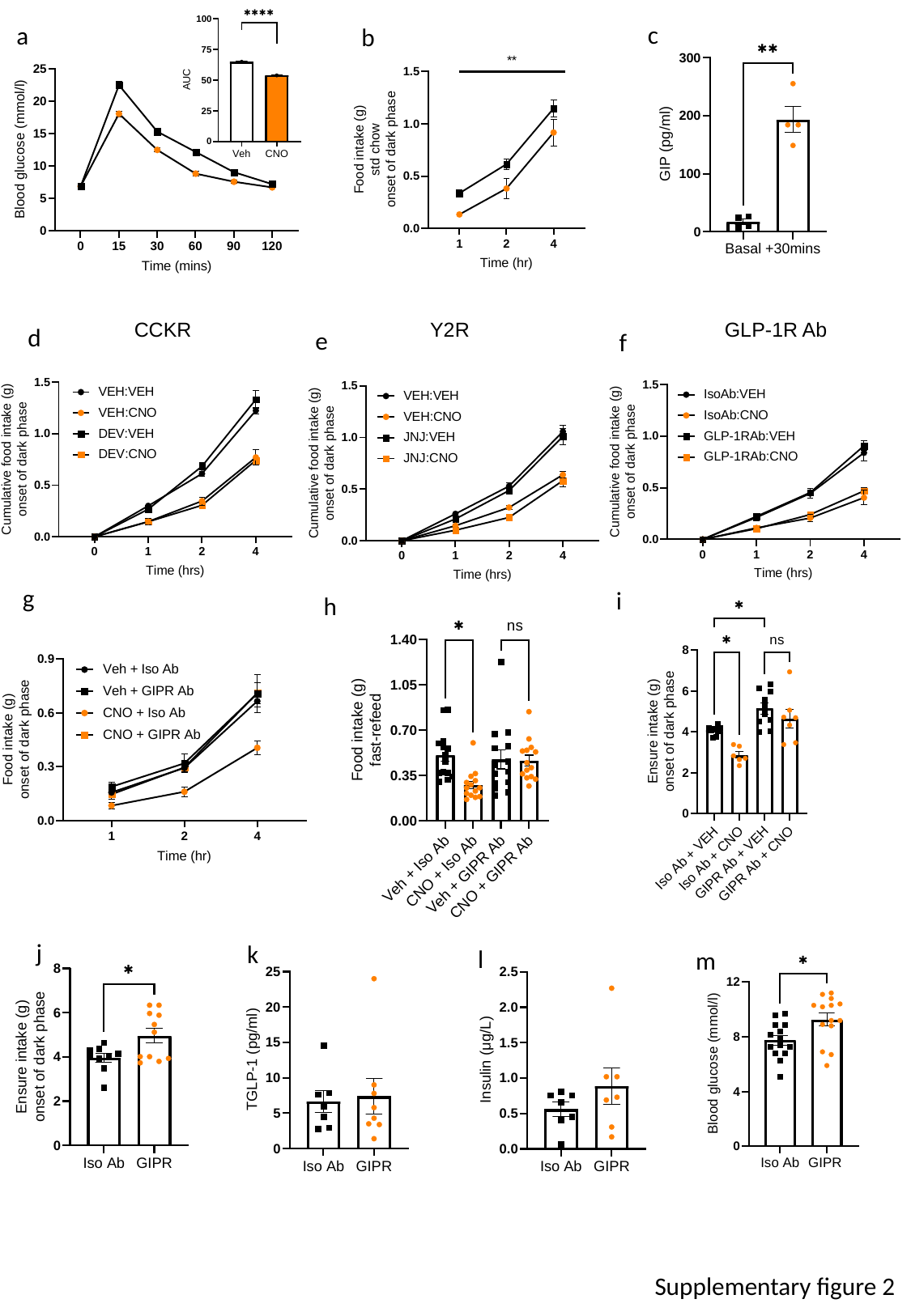

c
a
b
CCKR
GLP-1R Ab
Y2R
d
e
f
g
i
h
j
k
l
m
Supplementary figure 2

## Slide 3
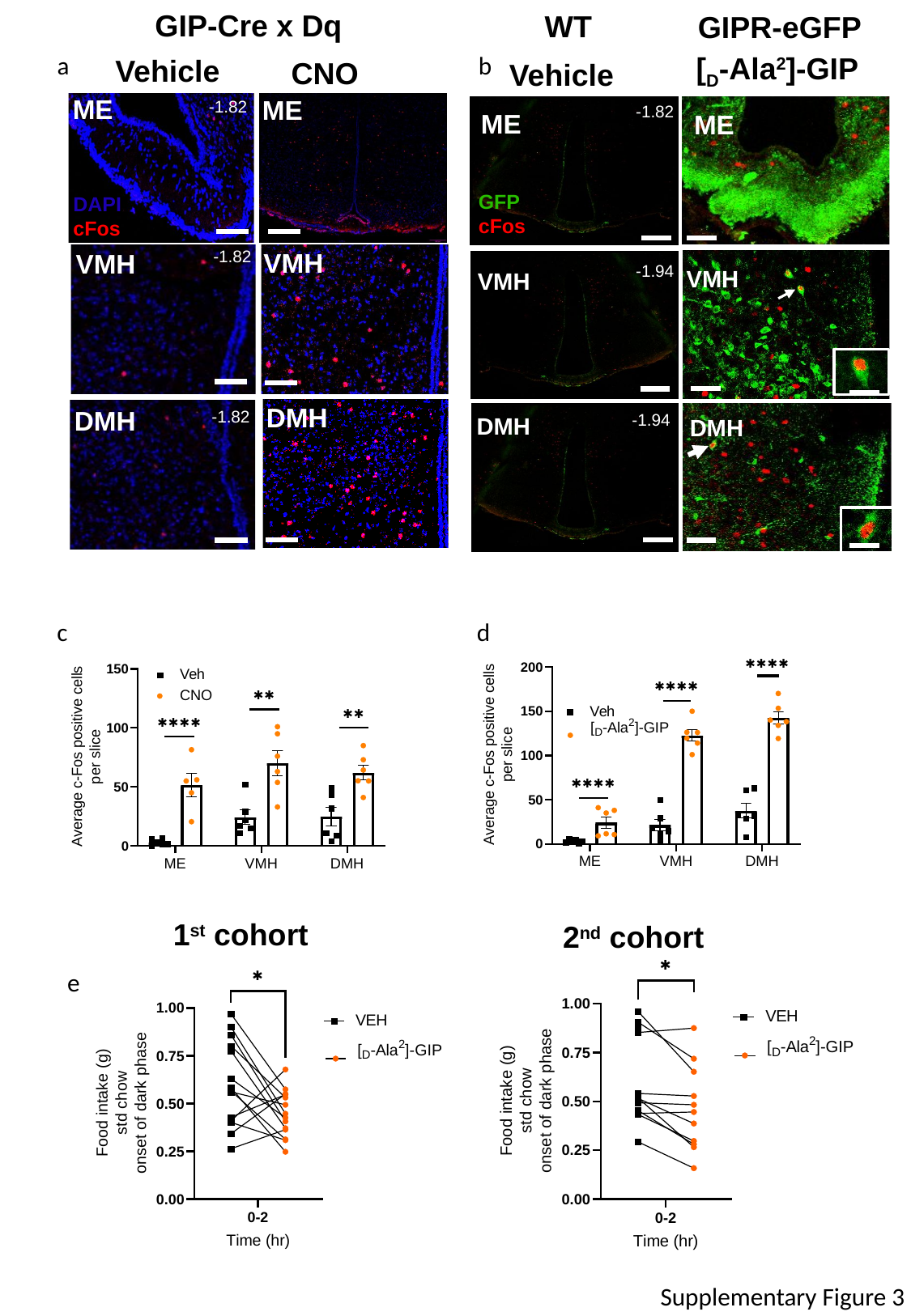

GIP-Cre x Dq
WT
GIPR-eGFP
[D-Ala2]-GIP
Vehicle
ME
ME
VMH
VMH
DMH
DMH
AP
AP
NTS
NTS
GFP
cFos
b
a
Vehicle
CNO
ME
VMH
DMH
AP
NTS
ME
VMH
ARH
DMH
AP
NTS
DAPI
cFos
-1.82
-1.82
-1.82
-1.94
-1.82
-1.94
c
d
AP
AP
NTS
NTS
1st cohort
2nd cohort
e
Supplementary Figure 3
